# Knockdown of PRDX2 sensitizes colon cancer cells to 5-FU by suppressing the PI3K/AKT signaling pathway

**DOI:** 10.1042/BSR20160447

**Published:** 2017-05-11

**Authors:** Jun Xu, Shouru Zhang, Rong Wang, Xingye Wu, Li Zeng, Zhongxue Fu

**Affiliations:** 1 Department of Gastrointestinal Surgery, The First Affiliated Hospital of Chongqing Medical University, Chongqing 400016, China; 2 Department of Gastrointestinal Surgery, Wuwei Tumor Hospital, Gansu 733000, China; 3 Department of Traditional Chinese Medicine, The First Affiliated Hospital of Chongqing Medical University, Chongqing 400016, China

**Keywords:** apoptosis, AKT, colon cancer, chemosensitivity, PRDX2, 5-FU

## Abstract

Although, 5-Fluorouracil (5-FU) remains widely used in adjuvant therapy in patients with colon cancer, resistance to 5-FU-based chemotherapy is an important reason for treatment failure. Recent studies have reported that an enhanced reactive oxygen species (ROS) scavenging system shows drug resistance to 5-FU. Peroxiredoxin-2 (PRDX2), is an important member of the ROS scavenging system, and may be a potential target that promotes chemosensitivity to 5-FU in colon cancer. Here, we depleted PRDX2 by PRDX2-shRNA-LV transduction in two colon cancer cell lines and found that *in vitro* PRDX2 knockdown facilitates cell death, and apoptosis in 5-FU-treated colon cancer cells. In addition, we found that PRDX2 depletion in mice treated with 5-FU resulted in, inhibition of tumor growth, compared with mice treated with 5-FU alone. Our data also suggested that the PI3K/AKT signaling pathway links PRDX2 with 5-FU-induced apoptosis in colon cancer. Furthermore, when PRDX2 was overexpressed in colon cancer cells, we found increased p-AKT protein expression and reduced Bcl-2/Bax protein expression. PRDX2 and p-AKT protein expression were analyzed by immunohistochemistry technology in human colon carcinoma tissues. Pearson correlation coefficient is 0.873 and *P*<0.05. PRDX2 depletion led to reduced p-AKT expression and PI3K/AKT pathway inhibition promoted cell apoptosis in HT29 cell line. Taken together, our study suggests that decreasing the expression of PRDX2 could be a promising strategy for increasing the sensitivity of colon cancer cells to 5-FU.

## Introduction

Colorectal cancer is the fifth most common cancer in China and one of the leading causes of cancer-related deaths worldwide [1]. Although, chemotherapy is very effective after surgery, resistance to drugs contributes to therapy failure [2]. Therefore, understanding the molecular mechanisms involved in drug resistance and identifying better targets to promote sensitivity to chemotherapeutics is of great importance.

5-Fluorouracil (5-FU) is one of the most widely used anticancer agents, which inhibits thymidylate synthetase and incorporates into both RNA and DNA. 5-FU induces tumor cell apoptosis by increasing the intracellular concentration of reactive oxygen species (ROS); however, an enhanced ROS scavenging system also shows drug resistance to 5-FU [3,4,5]. Peroxiredoxins (PRDX), belong to a family of antioxidant enzymes that protect cells from oxidative stress. PRDX2 is overexpressed in colon cancer and depletion of PRDX2 expression inhibits colon cancer cell growth [6,7]. These findings indicate that PRDX2 could be a potential target to address the problem of 5-FU resistance in colon cancer.

In our study, we used PRDX2-shRNA-LV to deplete PRDX2 expression in colon cancer cells. We showed that knocking down PRDX2 *in vitro* facilitates cell death and apoptosis in colon cancer cells treated with 5-FU. In addition, PRDX2 depletion *in vivo* in combination with 5-FU treatment, markedly inhibited tumor growth compared with treatment with 5-FU alone. Furthermore, we found that the PI3K/AKT signaling pathway plays a role in 5-FU-induced apoptosis in colon cancer. Therefore, decreasing the expression of PRDX2 could be a promising strategy for increasing the sensitivity of colon cancer cells to 5-FU.

## Materials and methods

### Cells and reagents

HT-29 and HCT116 human colon cancer cell lines were obtained from the Culture Collection of the Chinese Academy of Sciences (Shanghai, China). The cell lines were cultured in an RPMI 1640 medium (Gibco, U.S.A.) containing 10% FBS (PAN, Germany) and antibiotics (100 U/ml of penicillin and 100 μg/ml of streptomycin), in a humidified incubator maintained at 5% CO_2_ at 37°C.

Antibody against PRDX2 was purchased from Abcam plc (U.K.). Antibodies against Cleaved PARP were purchased from Cell Signaling Technology (Danvers, MA, U.S.A.). Antibodies against AKT1 were purchased from the Proteintech Group (Chicago, U.S.A.). Antibodies against p-AKT (Ser^473^) were purchased from Signalway Antibody LLC (College Park, MD, U.S.A.). MK-2206 2HCl was purchased from Selleck Chemicals (Houston, TX, U.S.A.).

### Transfection and stable cell line construction

Lentiviral constructs expressing PRDX2 shRNA (PRDX2-shRNA-LV) were purchased from Genechem (Shanghai, China). The PRDX2 shRNA vector sequence was as follows: forward: 5′-TCC TCT TTA TCA TCG ATG GCA ACT CGA GTT GCC ATC GAT GAT AAA GAG GTT TTT TC-3′; reverse: 3′-TCG AGA AAA AAC CTC TTT ATC ATC GAT GGC AAC TCG AGT TGC CAT CGA TGA TAA AGA GGA-5′. The positive experimental group (shPRDX2) consisted of PRDX2-shRNA-LV transduced into cells at a multiplicity of infection (MOI) of 60 using polybrene (10 μg/ml) and enhanced infection solution (Genechem, China). The negative control group (shCont) consisted of NC-shRNA-LV transduced into cells. After 72 h, cells that were transduced with the lentivirus containing shPRDX2 or shCont were selected with medium containing 5 μg/ml of puromycin. qRT-PCR and Western blotting were used to detect the inhibition rates of lentivirus-mediated shRNA targetting PRDX2.

### RNA extraction and qRT-PCR analysis

Cellular RNA was extracted from cells using the RNAiso plus reagent (Takara). RNA (1 μg) was reverse transcribed into cDNA with the PrimeScript™ RT Reagent Kit and gDNA Eraser (Takara). qRT-PCR was performed in an ABI Q6 qRT-PCR system (Applied Biosystems Inc., U.S.A.), according to the manufacturer’s instructions. The primers for PRDX2 were 5′-GCTGGGCTGTGAAGTGCTGG-3′ (forward) and 5′-ACGCCGTAATCCTCAGACAAGC-3′ (reverse), and B2M was used as an internal control. The primers for B2M were 5′-CTCTTTCTGGCCTGGAGGCTAT-3′ (forward) and 5′-AGTCAACTTCAATGTCGGATGGAT-3′ (reverse). All the reactions were performed in triplicates. The relative quantitation of gene expression was analyzed according to the ΔΔ*C*_t_method.

### *In vitro* cytotoxicity assay

Cells were seeded in 96-well plates at 1 × 10^4^ cells per well in RPMI 1640 medium with 10% FBS and treated with 0, 2.5, 5, 10, 20, 40 and 80 μg/ml of 5-FU (Cayman Chemical, Ann Arbor, Michigan, U.S.A.) for 48 h. The viability of cells was evaluated using the Cell Counting Kit-8 (CCK-8) assay (Dojindo Laboratories, Minato-ku, Tokyo, Japan), according to the manufacturer’s instructions.

### Cell apoptosis assay

Colon cancer cells were seeded in a culture flask at a density of 1 × 10^6^ cells and treated with 5-FU for 48 h. Then, the cells were stained with PE–conjugated Annexin V and 7-amino-actinomycin D (7-AAD) and apoptosis was detected using the Annexin V-PE Apoptosis Detection Kit (Beyotime, China). After staining and incubating, according to the manufacturer’s protocol, apoptosis was measured by flow cytometry (BD Biosciences, San Jose, CA, U.S.A.).

### Protein extraction and Western blot assay

Cells were lysed in RIPA lysis buffer (Beyotime, China) supplemented with PMSF (Beyotime, China). Cell lysates were cleared by centrifugation at 12000 rpm at 4°C for 20 min and collected. Protein concentration was determined by the BCA protein assay (Beyotime, China). Proteins were separated by SDS/PAGE (10% gel) and transferred on to PVDF membranes (Immobilon-P, Millipore, Germany). Membranes were blotted with the appropriate primary antibodies overnight at 4°C and then secondary antibodies for 1 h at room temperature. The antigen–antibody complexes were detected by ECL substrate (Advansta, California, U.S.A.).

### Immunohistochemical staining

All human colon carcinoma tissues were collected from colon cancer patients during surgery at the First Affiliated Hospital of Chongqing Medical University (Chongqing, China). The study was approved by the Ethics Committee of the First Affiliated Hospital of Chongqing Medical University (Chongqing, China). Informed consent was obtained for experiments with human subjects. Tissue samples were fixed in 4% paraformaldehyde, embedded in paraffin and processed as 5-μm thick sections. Sections were deparaffinized in xylene and rehydrated in graded ethanol. Antigen retrieval was performed by boiling in sodium citrate buffer (0.01 mmol/l). Endogenous peroxidases were inactivated with 3% H_2_O_2_, followed by incubation with goat serum for 20 min at 37°C, and the primary antibody overnight at 4°C. Finally, sections were incubated with the secondary antibody for 20 min at 37°C in a humidified chamber. Peroxidases bound to the antibody complex were visualized by treatment with 3,3′-diaminobenzidine chromogenic substrate solution. Immunolabeled sections were dehydrated in a series of graded ethanol and defatted in xylenes. The sections were then examined with an Olympus BX51 microscope (Olympus, Japan) under bright field illumination, and images were acquired with an Olympus DP70 camera (Olympus, Japan).

### Experiments with 5-FU treatment in nude mice

All studies involving animals were approved by the Ethics Committee of Chongqing Medical University. To establish a colon cancer mouse model, HCT116-shCont and HCT116-shPRDX2 cells (5 × 10^6^) in 0.2 ml PBS were injected intraperitoneally into the flanks of 4-week-old female BALB/c-nu mice (Animal Center of Chongqing Medical University, China). Tumors were allowed to grow for 5 days and then the animals were divided into four groups: shCont + PBS, shPRDX2 + PBS, shCont + 5-FU and shPRDX2 + 5-FU (each group: *n*=5). Five mice per group were injected intraperitoneally with either PBS or 5-FU (50 mg/kg per day, every 3 days, respectively). The survival of nude mice was measured on a regular basis.

### Statistical analysis

Statistical analyses were performed by using the SPSS software, version 21.0 (SPSS, Chicago, IL, U.S.A.) and GraphPad Prism 6. Correlation analyses were performed using the Pearson method. Data are presented as the mean ± S.D. from at least three independent experiments. Data were analyzed by Student’s *t* tests and the value of *P*<0.05 was considered significant.

## Results

### Lentivirus-mediated shRNA inhibition of PRDX2 in colon cancer cells

**Figure 1:**
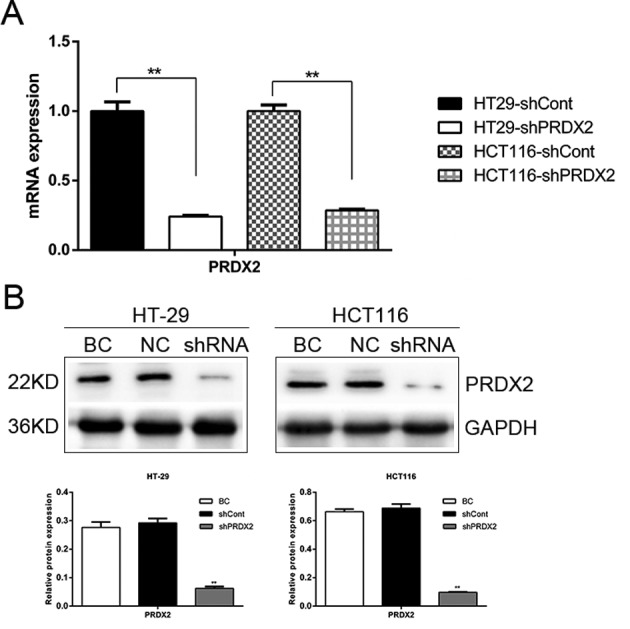
PRDX2 depletion in colon cancer cells (**A**) The mRNA levels of PRDX2 in shCont and shPRDX2 cells were analyzed by Q-PCR; **P<0.01. (**B**) The protein levels of PRDX2 in blank control (BC), shCont(NC) and shPRDX2 cells were analyzed by Western blotting; **P<0.01.

To investigate the role of PRDX2 in colon cancer cells, we transfected PRDX2-shRNA-LV and NC-shRNA-LV into the HT-29 and HCT116 cell lines. The mRNA and protein expressions of PRDX2 were significantly decreased in the shPRDX2 group compared with the shCont group (Figure 1A,B). These results demonstrate that the lentivirus-mediated shRNA targetted PRDX2 effectively and knocked down PRDX2 expression in colon cancer cells.

### PRDX2 depletion promotes cell death in colon cancer cells

**Figure 2:**
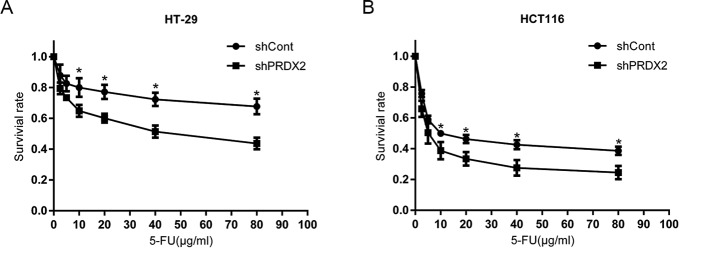
PRDX2 depletion promotes cell death in colon cancer cells (**A**) In HT-29 cells, the shCont group and shPRDX2 group were exposed to 5-FU for 48 h and cell viability was measured by the CCK-8 assay. (**B**) In HCT116 cells, the shCont group and shPRDX2 group were exposed to 5-FU for 48 h and cell viability was measured by the CCK-8 assay.

To determine the role of PRDX2 in colon cancer cells treated with 5-FU, we used the CCK-8 assay to detect the survival rate of colon cancer cells that were stably transfected with NC-shRNA-LV and PRDX2-shRNA-LV and treated with 5-FU at different concentrations for 48 h. We observed a lower survival rate in the shPRDX2 group, compared with the shCont group (Figure 2A,B). These results indicated that knocking down the expression of PRDX2 increased the chemosensitivity of colon cancer cells to 5-FU, in a dose-dependent manner.

### PRDX2 depletion promotes cell apoptosis in colon cancer cells

**Figure 3:**
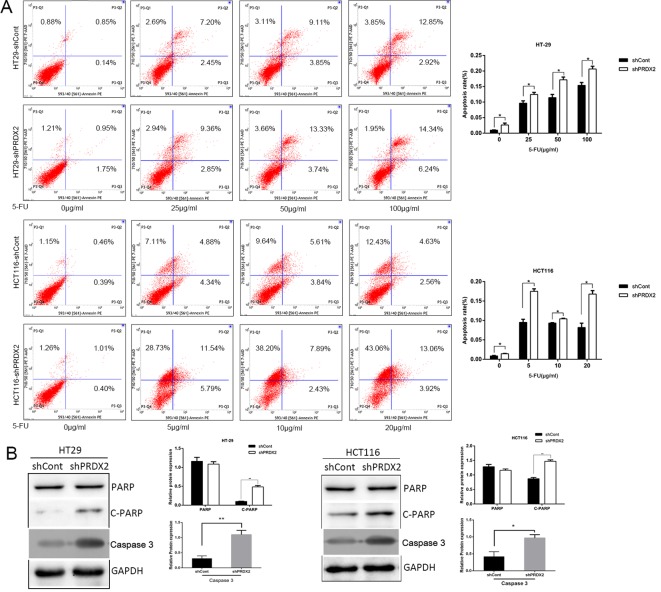
PRDX2 depletion promotes cell apoptosis in colon cancer cells (**A**) Cell apoptosis levels were evaluated by flow cytometry in HT-29 and HCT116 cells treated with 5-FU for 48 h; *P<0.05. (**B**) The protein levels of C-PARP and caspase-3 in HT-29 and HCT116 cells treated with 5-FU for 48 h were analyzed by Western blotting; *P<0.05, **P<0.01.

We analyzed the cell apoptosis by flow cytometry and found that knocking down PRDX2 expression in FU-treated colon cancer cells markedly increased apoptosis and protein levels of cleaved PARP and caspase-3 (Figure 3A,B). Collectively, our results suggest that PRDX2 depletion, combined with 5-FU, promotes cell apoptosis in colon cancer cells.

### PRDX2 depletion reduces colon cancer cell resistance to 5-FU *in vivo*

**Figure 4:**
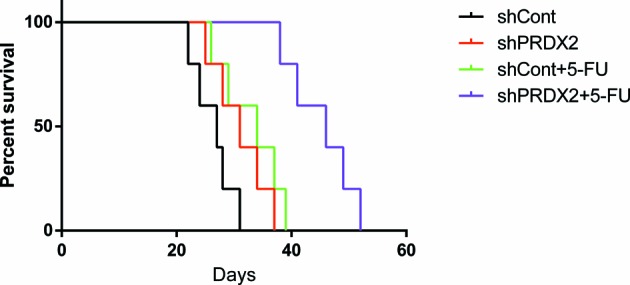
PRDX2 depletion reduces the resistance of colon cancer cells to 5-FU *in vivo* Five female BALB/c-nu mice were placed in each group. The shCont group treated with 5-FU and shPRDX2 group with PBS injection had longer survival times than nude mice in the shCont group with PBS injection. However, nude mice in the shPRDX2 group with 5-FU treatment had the longest survival time compared with the other three groups; *P<0.05.

To ascertain the effect of PRDX2 knockdown in colon tumor growth, we examined the *in vivo* efficacy of 5-FU in mice bearing abdominal tumors that originated from HCT116-shPRDX2 or HCT116-shCont cells. Using Kaplan–Meier analysis with the log-rank test, we found that the mice in the shCont group treated with 5-FU and the untreated shPRDX2 group had longer survival times than the mice in the untreated shCont group. However, the mice in the shPRDX2 group treated with 5-FU exhibited the longest survival time of the four groups (Figure 4). These *in vivo* results suggest that PRDX2 contributes to anticancer drug resistance and that decreasing the expression of PRDX2 in combination with 5-FU treatment reduces tumor development in colon cancer.

### Expression of PRDX2 and p-AKT in human colon carcinoma

**Figure 5:**
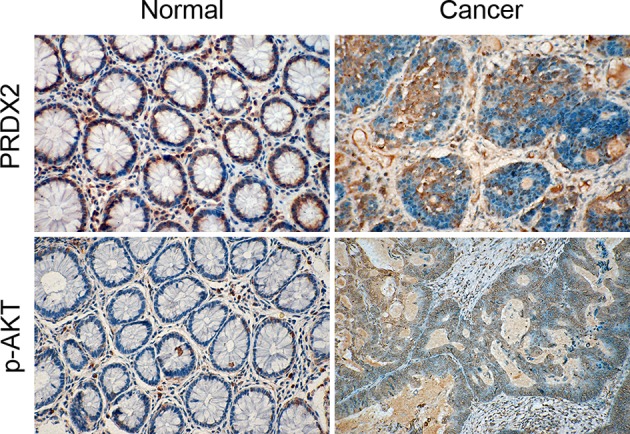
The expression of PRDX2 and p-AKT in human colon carcinoma Representative images of PRDX2 and p-AKT expression in human colon carcinoma samples and normal adjacent tissues are shown.

To investigate whether PI3K/AKT contributes to PRDX2-induced drug resistance in colon cancer, we used immunohistochemistry to detect PRDX2 and p-AKT protein levels in tumor tissues and their corresponding normal colorectal mucosal tissues from 50 colon cancer patients. We found that PRDX2 and p-AKT were strongly expressed in the cytoplasm of colon cancer cells, and were weakly expressed in normal colon mucosal tissues (Figure 5). The proportion of PRDX2-positive cells and p-AKT-positive cells were 86% (43/50) and 88% (44/50), respectively. On further analysis, we found that PRDX2 and p-AKT showed a significant positive correlation (*r* =0.738, *P*<0.05) through Pearson correlation (Table 1). These results demonstrate that PI3K/AKT contributes to PRDX2-mediated 5-FU resistance.

**Table 1:** Correlation of PRDX2 and p-AKT expression in colon cancer tissue samples

p-AKT	PRDX2		Pearson correlation coefficient	*P*-value
	Positive (*n*=44)	Negative (*n*=6)		
Positive (*n*=43)	42	1	0.738	<0.05
Negative (*n*=7)	2	5		

### PI3K/AKT is crucial for PRDX2-mediated 5-FU resistance in colon cancer

**Figure 6:**
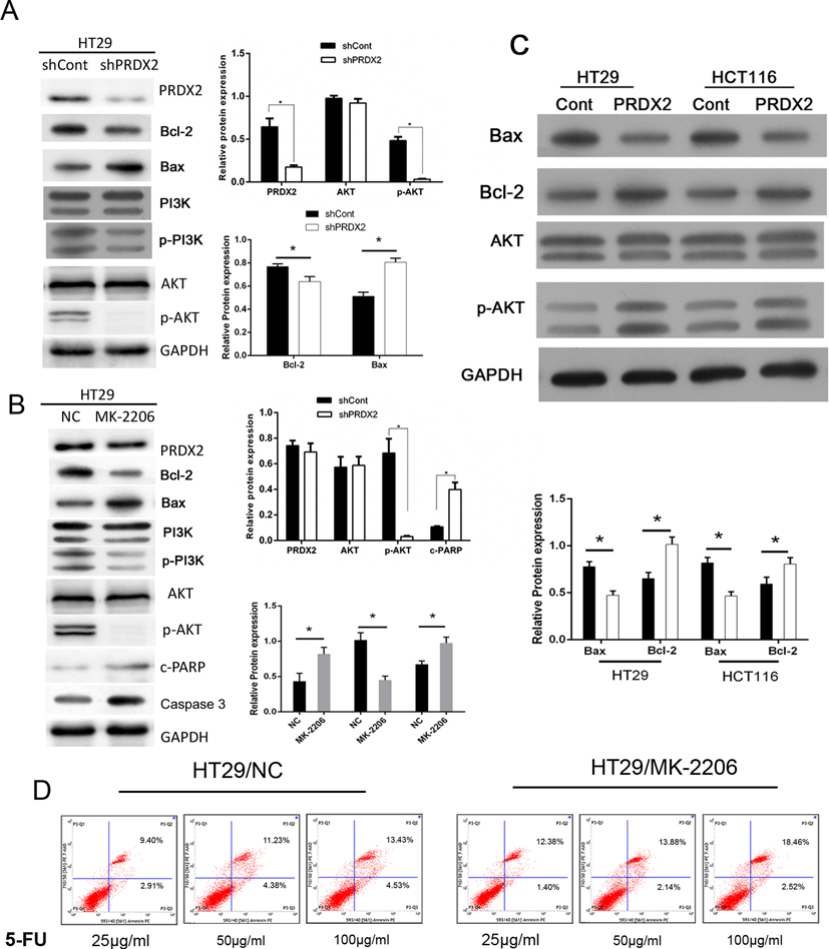
PI3K/AKT is crucial for PRDX2-mediated 5-FU resistance in colon cancer (**A**) The protein expressions of PRDX2 AKT/PI3K, Bax/Bcl-2 were analyzed by Western blotting in HT-29-shCont or HT-29-shPRDX2 cells; *P<0.05. (**B**) HT29 and HT116 cells were treated with LV-PRDX2 and the protein expressions of AKT, p-AKT, Bax and Bcl-2 were detected by Western blotting. (**C**) HT-29 cells were treated with MK-2206 2HCl for 48 h and then the protein expressions of PRDX2, AKT/PI3K, Bax/Bcl-2 were detected by Western blotting; *P<0.05. (**D**) Cell apoptosis levels were evaluated by flow cytometry in HT-29 cells treated with and without MK-2206 2HCl; *P<0.05.

The protein expressions of AKT, p-AKT and p-PI3K were analyzed by Western blotting in HT-29-shCont or HT-29-shPRDX2 cells. We found that depleting PRDX2 resulted in reduced p-AKT and p-PI3K expressions (Figure 6A). Furthermore, as shown in Figure 6B, 5-FU-treated HT29 and HCT116 cells were infected with overexpressed PRDX2, LV-PRDX2, and protein expressions of p-AKT and Bcl-2/Bax protein were observed. To verify the role of PI3K/AKT in PRDX2-induced drug resistance, we treated HT-29 cells with the AKT inhibitor, MK-2206 2HCl and then observed the resistance of HT-29 cells to 5-FU. MK-2206 inhibited AKT/PI3K protein expression. However, elevated expressions of caspase-3 and Bax were detected, along with suppressed Bcl-2 expression post MK-2206 treatment (Figure 6C). These results were validated by measuring apoptosis by using flow cytometry after cells were treated 5-FU in the presence or absence of MK-2206 HCl. We observed higher C-PARP and Bax protein expression and a higher rate of apoptosis in colon cancer cells treated with MK-2206 2HCl (Figure 6C,D). Together, these data suggest that 5-FU targets PRDX2 and down-regulates its expression through the AKT/PI3K pathway.

## Discussion

In general, higher levels of ROS contribute to tumor progression and development in most tumors compared with normal tissues [8]. Moreover, oxidative stress is associated with the efficacy of cancer treatments, including chemosensitivity and apoptosis [9]. However, extremely high ROS concentrations are deadly to tumor cells [10]. 5-FU inhibits thymidylate synthetase and incorporates into both RNA and DNA, inducing intracellular increases in O^2−^ levels in colon cancer [5,11]. This results in a new redox balance with a higher ROS level and an enhanced ROS scavenging system, that is resistant to 5-FU [12]. For these tumors, combining ROS generating drugs and restraining the cellular antioxidant capacity is effective. For example, combining arsenic trioxide and ascorbic acid, induces GSH depletion, and is effective in treating relapsed or refractory multiple myeloma [13]. In fact, PRDX3 knockdown combined with 5-FU increased cell death and tumor suppression in colon cancer [14].

PRDX2, is an important member of the ROS scavenging system, and exerts an anti-apoptotic effect on colon cancer cells. Lu et al. [15] showed that PRDX2 is up-regulated and protects cells from oxidative stress in colorectal cancer. In our study, we found that knockdown of PRDX2 expression promotes sensitivity to 5-FU in colon cancer cells. These results suggest that depleting PRDX2 combined with 5-FU treatment could be a promising strategy for anticancer drug resistance.

Hwang et al. [4] found that 5-FU treatment induced ROS generation in human lung carcinoma cells, leading to a higher level of antioxidant enzymes and resistance to 5-FU. PRDX proteins protect MCF-7 breast cancer cells from doxorubicin-induced toxicity [16]. PRDX6 overexpression attenuated cisplatin-induced apoptosis in human ovarian cancer cells [17]. Kalinina et al. [18] found that cisplatin resistance in cancer cells was accompanied with a significantly increased expression of *PRDX2* gene. In pancreatic cancer, a proteomic study using gemcitabine-sensitive KLM1 and -resistant KLM1-R cells showed that PRDX2 was significantly up-regulated in KLM1-R cells [19]. The expression of PRDX2 was higher in osteosarcoma patients that were poor responders to induction chemotherapy. In addition, PRDX2 depletion contributed to increased sensitivity to chemotherapeutic drugs in osteosarcoma cells [20,21]. Furthermore, PRDX2 localized to the nucleus and PRDX2 depletion markedly promoted cell death in cancer cells treated with DNA damaging agents [22]. These observations are consistent with data from our study. We treated shCont and shPRDX2 transfected colon cancer cells with 5-FU and found that apoptotic cells, C-PARP and caspase-3 protein expression increased markedly when PRDX2 was depleted. These results indicated that PRDX2 depletion promotes sensitivity to 5-FU in colon cancer cells.

Recent studies revealed that the PI3K/AKT pathway plays an important role in chemoresistance [23]. NPC (nasopharyngeal carcinoma) cell lines stably overexpressing miR-3188 exhibited significantly increased sensitivity to 5-FU by inactivating the (PI3K)/AKT pathway [24]. NECTIN-4 increased the resistance of colon cancer cells to 5-FU by inducing the PI3K/AKT cascade [25]. Activation of the PI3K/AKT pathway contributes to resistance to multiple cancer therapies, and is deemed a poor prognostic factor for cancers [26]. In our study, we found that p-AKT protein levels were lower in PRDX2-depleted colon cancer cells. Moreover, when the AKT inhibitor, MK-2206 was added to 5-FU treated colon cancer cells, we oserved higher C-PARP protein levels and increased cell apoptosis. These results indicated that PI3K/AKT is a key pathway that contributes to PRDX2-mediated 5-FU-induced apoptosis.

Taken together, our results demonstrate that inhibiting PRDX2 expression promotes 5-FU-induced apoptosis in colon cancer cells via the PI3K/AKT signal pathway.

## Abbreviations

CCK-8: cell counting kit-8
PRDX2: peroxiredoxin-2
ROS: reactive oxygen species
5-FU: 5-Fluorouracil
LV: lentivirus
